# Evidence for Structural and Functional Damage of the Inner Retina in Diabetes With No Diabetic Retinopathy

**DOI:** 10.1167/iovs.62.3.35

**Published:** 2021-03-24

**Authors:** Giovanni Montesano, Giovanni Ometto, Bethany E. Higgins, Radha Das, Katie W. Graham, Usha Chakravarthy, Bernadette McGuiness, Ian S. Young, Frank Kee, David M. Wright, David P. Crabb, Ruth E. Hogg

**Affiliations:** 1 Optometry and Visual Sciences, City, University of London, London, United Kingdom; 2 NIHR Biomedical Research Centre, Moorfields Eye Hospital NHS Foundation Trust and UCL Institute of Ophthalmology, London, United Kingdom; 3 Centre for Public Health, Queen's University Belfast, Block B, Royal Hospital, Belfast, Northern Ireland

**Keywords:** diabetes, retinal ganglion cells, microperimetry, OCT, spatial summation

## Abstract

**Purpose:**

To provide structural and functional evidence of inner retinal loss in diabetes prior to vascular changes and interpret the structure-function relationship in the context of an established neural model.

**Methods:**

Data from one eye of 505 participants (134 with diabetes and no clinically evident vascular alterations of the retina) were included in this analysis. The data were collected as part of a large population-based study. Functional tests included best-corrected visual acuity, Pelli-Robson contrast sensitivity, mesopic microperimetry, and frequency doubling technology perimetry (FDT). Macular optical coherence tomography volume scans were collected for all participants. To interpret the structure-function relationship in the context of a neural model, ganglion cell layer (GCL) thickness was converted to local ganglion cell (GC) counts.

**Results:**

The GCL and inner plexiform layer were significantly thinner in participants with diabetes (*P* < 0.05), with no significant differences in the macular retinal nerve fiber layer or the outer retina. All functional tests except microperimetry showed a significant loss in diabetic patients (*P* < 0.05). Both FDT and microperimetry showed a significant relationship with the GC count (*P* < 0.05), consistent with predictions from a neural model for partial summation conditions. However, the FDT captured additional significant damage (*P* = 0.03) unexplained by the structural loss.

**Conclusions:**

Functional and structural measurements support early neuronal loss in diabetes. The structure-function relationship follows the predictions from an established neural model. Functional tests could be improved to operate in total summation conditions in the macula, becoming more sensitive to early loss.

Diabetic retinopathy (DR) is the leading cause of blindness worldwide in working-age adults.^1^^–^^3^ The role of vascular damage and new vessel proliferation is widely recognized and is ultimately responsible for the loss of sight.^1^^,^^3^ However, recent evidence suggests that direct retinal neuronal damage in diabetic patients might precede evident changes to the retinal blood vessels^4^ and be a risk factor for progression to DR.^5^ The damage mainly manifests as retinal ganglion cell (RGC) loss through apoptosis, resembling other neurodegenerative diseases.^6^^–^^8^

Both functional and structural evidence has been provided to support RGC loss in diabetic patients. Several imaging studies, earlier with scanning laser polarimetry^9^^–^^11^ and more recently with spectral domain optical coherence tomography (SD-OCT), observed thinning of the ganglion cell layer (GCL), retinal nerve fiber layer (RNFL), and inner plexiform layer (IPL) in patients with minimal or no vascular diabetic retinopathy.^4^^,^^12^^–^^17^ A wide array of tests have also been employed to detect the functional implications of such structural changes. Besides visual acuity, reported in almost all studies, functional tests have included Pelli-Robson contrast sensitivity,^18^^–^^20^ microperimetry,^20^^–^^24^ Rarebit perimetry,^18^^,^^25^^,^^26^ frequency doubling technology perimetry (FDT),^18^^,^^19^^,^^27^^–^^29^ standard automated perimetry (SAP),^18^^,^^28^^–^^31^ and quick contrast sensitivity function (qCSF).^18^ All these functional assessments have been able to show, to a different extent, some degree of functional impairment in patients with diabetes mellitus (DM) with no or minimal DR when compared to people without DM. Microperimetry is particularly appealing for its high spatial accuracy and has been used for topographical structure-function mapping of early diabetic damage.^20^^,^^22^ FDT perimetry has also shown promising results in this context and is particularly valuable for its sensitivity to inner retina damage.^19^^,^^32^ Jackson et al.,^19^ Parravano et al.,^29^ Joltikov et al.,^18^ and Bao et al.^27^ all reported reduced sensitivity to FDT stimuli in patients with early or no DR.

Many of these effects in visual function are, however, subtle and difficult to identify. Although structural analyses on large databases of OCT scans exist,^16^^,^^17^ functional tests have been performed on much smaller cohorts, especially when considering diabetic people with no vascular damage. The only large-scale functional testing results in people with DM and no DR come from a screening survey performed in the United States^27^ with a suprathreshold FDT test. The study confirmed the usefulness of FDT as an indicator of early inner retinal damage in diabetes. However, the absence of measured sensitivity thresholds and structural data prevented a comprehensive quantification of the structural and functional damage and their relationship.

In this work, we use prospectively collected structural (SD-OCT) and functional data from a large cohort of healthy people and diabetic patients with no DR to characterize early neuronal damage in diabetes. The data are part of a population-based collection, the Northern Ireland Sensory Ageing (NISA) study (https://clinicaltrials.gov/ct2/show/NCT02788695), conducted at Queen's University Belfast (QUB). The functional data include best-corrected visual acuity (BCVA), Pelli-Robson log_10_ contrast sensitivity (PR-logCS), microperimetry, and FDT threshold perimetry. We use these data to test the hypothesis that structural and functional damage of the inner retina is present in DM prior to clinically evident vascular changes.

## Methods

### Data Collection

The NISA study (https://clinicaltrials.gov/ct2/show/NCT02788695) is a follow-up to the NICOLA (Northern Ireland Cohort for the Longitudinal Study of Ageing) study (https://www.qub.ac.uk/sites/NICOLA/), a prospective population-based study of early imaging and functional biomarkers of DR and age-related macular degeneration (AMD). The selection steps are illustrated in a flowchart in the Appendix. The NICOLA study involved a computer-assisted home interview followed by a health assessment at the Northern Ireland Clinical Research Facility (NICRF), including an evaluation of eye health. People from the NICOLA cohort with at least one of the following characteristics were invited for the NISA follow-up data collection: (1) no retinal diseases, (2) self-reported diagnosis of DM either during the home interview or the health assessment, and (3) early or intermediate AMD. The sample of diabetic people (type I or type II) was then extended with patients recruited directly from the Belfast Trust Diabetic Retinopathy Hospital Clinics at QUB. Participants underwent BCVA test with an Early Treatment Diabetic Retinopathy Study (ETDRS) chart, PR-logCS test with a Pelli-Robson chart, microperimetry and FDT perimetry (described in detail in the following paragraphs), an SD-OCT scan (described in detail in the next paragraph), fundus color picture (CX-1 Digital Fundus Camera; Canon U.S.A., Inc, Tokyo, Japan), color ultra-wide-field imaging (UWFI) images centered on the fovea (Optomap Panoramic 200Tx scanning laser ophthalmoscope; Optos PLC, Dunfermline, Scotland, UK), and a measurement of axial length (AL; Lenstar LS 900 Biometer, Haag-Streit AG, Köniz, Switzerland). Lens opacity in phakic eyes was graded with a Pentacam Scheimpflug System (Oculus, Wetzlar, Germany) using the Pentacam Nucleus Staging (PNS) classification.^33^ All imaging was performed after pharmacologic dilation with tropicamide 1%. For all participants, the eye with better BCVA was selected for the study, choosing at random if they were both eligible.

Fundus color pictures for all participants were classified by two graders (authors RD and UC) to identify signs of AMD (Beckman classification)^34^ and DR. Disc and macula color images and UWFI were assessed for characteristic DR features in the central and peripheral retina and then staged using the national screening for DR system for England and Wales into four levels: none (R0), background (R1), preproliferative (R2), and proliferative (R3). Participants not recruited from the DR clinic were identified as diabetic if they self-reported a diagnosis of DM. The duration of the disease was also recorded when provided. All participants over 50 years of age and all diabetic patients were also invited to have a blood sample taken to measure the concentration of plasma glycated hemoglobin (HbA1C). Participants with no record of diabetes were classified as diabetic if the HbA1C was ≥6.5%. Refusal to have the blood sample taken did not prevent inclusion.

Only healthy participants (starting *n* = 406) or diabetic patients classified as R0 (starting *n* = 159) were considered for this analysis. Eyes with intermediate or advanced AMD were also excluded (*n* = 17). People with signs of early AMD were included in the analysis to avoid overselection of participants, especially for the no-DM cohort. Of the remaining 548, we only selected people for whom either microperimetry or FDT was available (*n* = 545). Hence, the following selection steps were applied to a starting sample of 395 healthy participants and 150 diabetic patients.

### SD-OCT Scans

**Figure 1.**
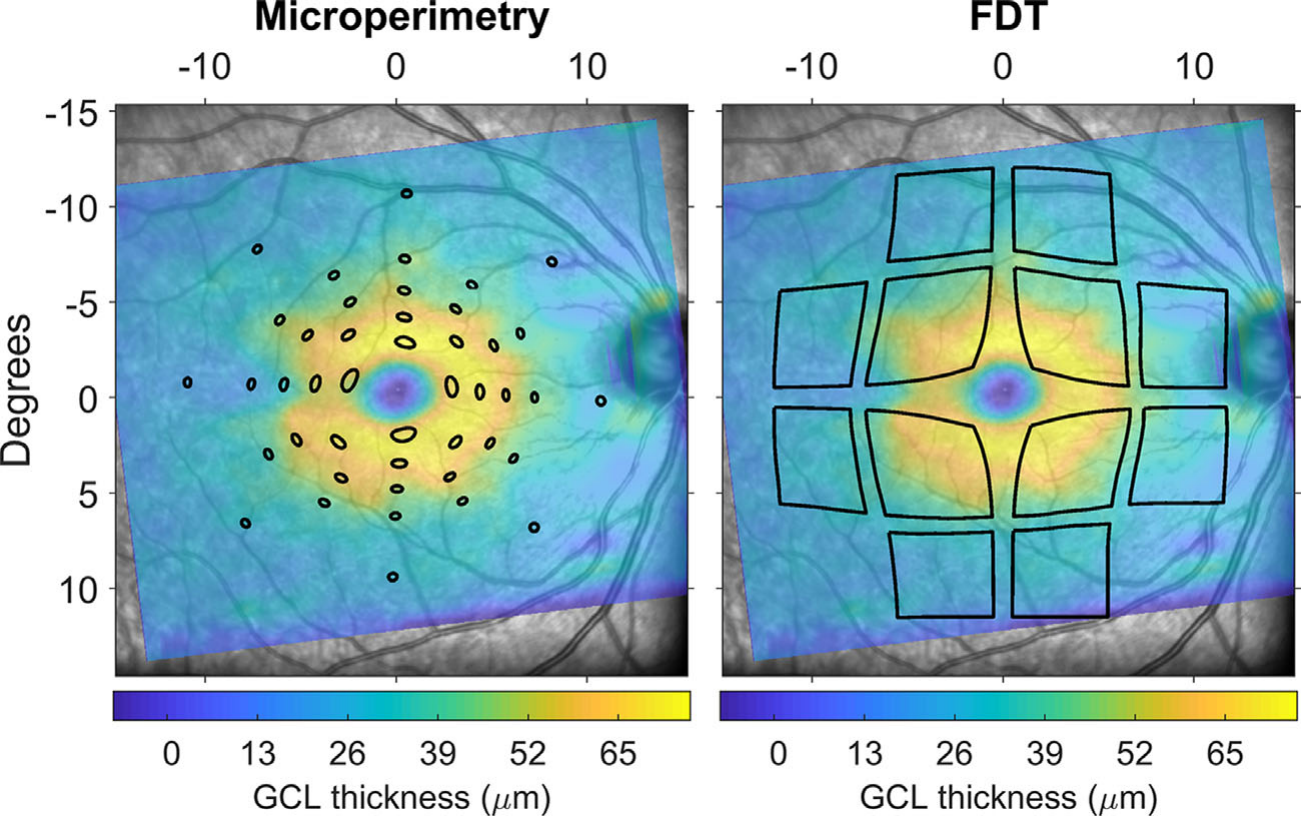
Schematic of the stimulus displacement applied for the topographic structure-function analysis, overlaid to the GCL thickness map from the Spectralis SD-OCT. Areas enclosed within the *black lines* were used for calculations. Microperimetric stimuli were mapped by aligning the fundus images from the MAIA and the Spectralis. FDT stimuli were centered on the fovea.

Structural data were collected using a Spectralis SD-OCT (Heidelberg Engineering, Heidelberg, Germany). A macular volume scan was acquired for each study eye. The volume was composed of 61 horizontal B-scans (ART 9) covering a rectangular patch 30 × 25 degrees tilted by 7 degrees (counterclockwise for right eyes, clockwise for left eyes) to match the average inclination of the fovea-disc axis (Fig. 1). The scans were tracked on the retina using a scanning laser ophthalmoscope (SLO) that continuously captured an infrared fundus picture to compensate for eye movements. A circumpapillary RNFL (cp-RNFL) scan was also collected for the study eye. All images were evaluated by two graders (authors RD and UC) to identify poor-quality scans and manually correct the segmentations where necessary. Poor quality was defined as the inability to accurately identify all retinal layers either because of low signal strength or because of partially or totally out-of-frame scans. An ophthalmologist (GM) visually inspected all scans from eyes that matched the inclusion criteria (described above) to identify people with vitreo-retinal alterations (such as vitreo-retinal tractions or epiretinal membranes), focal loss of the inner retina on macular scans or of the RNFL on the cp-RNFL scans (either from vascular occlusions or possible glaucoma), and diffused advanced cp-RNFL thinning and/or optic nerve head (ONH) cupping likely attributable to glaucomatous damage. Standard white-on-white perimetry and a measurement of the intraocular pressure were not obtained for this data collection. Hence, a careful selection based on structural criteria was necessary. A total of 14 eyes were excluded due to poor quality of the scans, 10 due to the presence of vitreo-retinal diseases, and 16 due to focal inner retinal loss or possible glaucoma. The same criteria were applied to cp-RNFL scans for participants whose macular scan was included, leading to the exclusion of 119 of 505 scans (14 from diabetic patients), all because of poor quality. The absence of a viable cp-RNFL did not prevent inclusion in the analysis. Hence, selection was only based on the macular scans. For the final selection of scans, the median (interquartile range) quality index was 30.7 (28.9, 32.5) dB for the macular scans and 29.3 (23, 34.7) dB for the cp-RNFL scans.

#### Microperimetry

Microperimetric data were collected using a MAcular Integrity Assessment device (MAIA; CenterVue, Padua, Italy). The MAIA performs continuous infrared imaging of the retina to track and compensate for eye movements occurring during the test.^35^^–^^37^ The test was performed in mesopic conditions (1.27 cd/m^2^ background illumination) with an achromatic stimulus (0.43 degrees diameter) using a 4-2 staircase strategy. At the beginning of the test, a 10-second fixation trial was used to locate the preferred retinal locus (PRL) of fixation, used as the center of the perimetric grid. Notice that this might not coincide with the anatomical fovea. All tests were preceded by a training phase with the “fast” protocol to minimize learning effect. The grid was composed of 44 locations distributed on five concentric rings at 1, 2.3, 4, 6, and 10 degrees of eccentricity from the PRL. No exclusion was performed based on fixation metrics or blind spot responses since, given the use of fundus tracking, these metrics are unlikely to be related to the reliability of the test. Furthermore, in a previous analysis, we have shown both these metrics to be poor predictors of test-retest variability in microperimetry.^38^

#### FDT Perimetry

FDT data were collected with a Matrix device (Zeiss Meditec, Dublin, CA, USA). The test was performed with the 24-2 threshold program. The stimuli were 5 degree squares with a vertical sine wave grating (0.5 cycles/degree) counterphase flickered at 18 Hz. The threshold was measured using a Zippy Estimation through Sequential Testing (ZEST)^39^ strategy. Only one eye per participant was tested (the same as microperimetry when both were performed). In contrast to microperimetry and standard SAP, Matrix FDT uses Michaelson's definition of contrast instead of Weber's.^40^ In the context of sinusoidal grating stimuli, they are, however, equivalent.^41^ Importantly, the Matrix FDT defines the sensitivity scale so that change of one log_10_ unit of contrast corresponds to 20 dB instead of 10 dB, as in microperimetry and SAP.^40^ Two tests were excluded due to high false-positive errors (>33%).

### Data Analysis

#### Structural Metrics

All OCT data were exported as RAW files (.vol) using the Heidelberg Eye Explorer. The files were then read in MATLAB (MathWorks, Natick, MA, USA) using a custom-made code. The segmentations were used to generate thickness maps for the whole retina, the RNFL, the GCL, the IPL, and the outer retina (from the outer limit of the IPL to the Bruch's membrane). The maps were interpolated and smoothed to match the size of the reference infrared fundus image (768 × 768 pixels, 30 × 30-degree field of view), padding with zeros where the OCT data were missing (i.e., outside the scanning pattern). The interpolation was performed using a thin plate spline (*tpaps* function in MATLAB) with anisotropic smoothing parameters, so that smoothing was stronger across B-scans than within a B-scan. The fovea was automatically identified through template matching. Correct detection was confirmed through visual inspection.

Topographic average thickness values for all layers were measured using a standard ETDRS grid, with three concentric rings of 1 mm, 3 mm, and 6 mm external diameter. The two outermost rings were divided into four sectors (superior, inferior, nasal, and temporal). The size of the grid was corrected for AL using our implementation of a schematic eye^42^ proposed by Drasdo and Fowler.^43^ The statistical analysis was performed in R (R Foundation for Statistical Computing, Vienna, Austria) using a linear mixed-effect model with a random intercept to account for multiple measurements (different sectors) from the same eye. The response variable was the measured thickness, and the model included a categorical fixed effect for the group (either healthy or diabetic), a categorical fixed effect for the sector, the interaction between the two fixed effects, and age (years) as a continuous predictor. The *P* values were corrected for multiple comparisons (*n* = 9 tests) with the Bonferroni-Holm method.^44^ Finally, global differences between the two groups across all sectors were tested for each layer by setting the sector as a crossed random effect. Global age-corrected differences are reported as estimate (95% confidence interval [CI]). The α level was 0.05 for all analyses.

The cp-RNFL scans were corrected for ocular magnification using the formula provided by Kang et al.^45^ However, such a compensation and other similar methods proposed introduce a positive correlation with AL, which then needs to be accounted for in the analysis.^45^^,^^46^ Therefore, when analyzing the cp-RNFL scans, we included AL as a covariate, together with age.

#### Functional Metrics

Microperimetric and FDT data were exported as XML files and read in MATLAB. The mean sensitivity (MS) for MAIA tests was calculated excluding the foveal location, which was not used for the structure-function analysis (see later). The Matrix FDT provides a calculation of the global mean deviation (MD), pattern standard deviation (PSD), and values for the pointwise sensitivity, total deviation (TD), and pattern deviation (PD). We additionally calculated the global MS as the average of the 52 locations within the 24-2 grid, excluding the two blind spot locations. Since our main analysis focused on the macular region, we only used pointwise data for the 12 central locations (within 10 degrees from fixation). Global indices for the macula were also calculated as the average of the 12 corresponding central TD values (central mean deviation, cMD) and the central sensitivity values (central mean sensitivity, cMS). All statistical comparisons, including for BCVA and PR-logCS, were performed with simple multivariate linear models with age as a covariate, except for the MD and the cMD, which already account for normal aging. BCVA was converted from letter counts to log_10_ minimum angle of resolution (logMAR) for analysis.

#### Structure-Function Relationship

For our main structure-function analysis, we focused on the GCL, assumed to be mostly composed of the bodies of the RGC neurons. The GCL thickness maps were transformed into estimates of local RGC density using the method proposed by Raza and Hood^47^ and based on the histology maps by Curcio and Allen.^48^ The maps were corrected for axial length assuming a global expansion model, as previously described.^42^ These density maps can then be used to derive customized local or global RGC counts.

##### Global Structure-Function Relationship

The global structure-function relationship was studied using the MS for the MAIA and the cMD and cMS for the FDT. The functional metric was used as the response variable. The structural parameter was the total number of RGCs within 12 degrees from the fovea, covering the area tested by both the 12 central FDT and the MAIA grid after accounting for RGC displacements (see next paragraph). The MS for the MAIA and FDT tests was analyzed using a multivariate model that included age as a covariate. The RGC counts were log_10_ transformed prior to analysis to match the scale of perimetric data. This is also a widely applied method to relate RGC counts to perimetric sensitivities^42^^,^^49^^–^^51^ (see also the Appendix). A secondary analysis of the correlation between global FDT MS with the average cp-RNFL (corrected for ocular magnification^45^) thickness was also performed, with age and AL as covariates.

##### Topographic Structure-Function Relationship

We used pointwise data to explore the local structure-function relationship at different eccentricities. The structural metric was the local RGC count corresponding to each location. We accounted for RGC displacement in the macular region using our generalized implementation^42^ of the model proposed by Drasdo et al.^52^ Instead of simply displacing the center of the tested locations, we displaced the whole perimeter of the stimuli, since we have previously proven this to be the correct method to obtain accurate structurally derived RGC counts^42^ (Fig. 1).

Since the same RGC count could produce a different psychophysical sensation (i.e., sensitivity) at different eccentricities, especially in the perifoveal region, our structure-function models used a categorical fixed effect to account for eccentricity. An interaction term with the log_10_(RGC counts) also allowed for a different slope for each eccentricity. Finally, an interaction term between the eccentricity fixed effect and the group fixed effect (healthy or diabetic) allowed for formal testing of statistically significant differences in slopes between the two groups, which would indicate different change in sensitivities with the same change in RGC count. A more detailed explanation of the model and its interpretation is given in the Appendix. For FDT data, the categorical fixed effect identified each one of the 12 central locations as a separate level. For the MAIA, locations were instead grouped by their eccentricity from the PRL. Prior to analysis, the fundus image from the MAIA was matched with the SLO fundus picture from the Spectralis SD-OCT using an affine or projective transformation, so that the tested locations could be accurately reported onto the structural maps. The alignment was performed in MATLAB and visually inspected (GM) to ensure it was correct. When incorrect, the alignment was repeated by placing manual landmarks on the two images. A satisfactory alignment could be obtained for all the included OCT/MAIA pairs. Manual intervention was required for 327 of 486 alignments.

## Results

### Sample Description

Descriptive statistics of the selected sample are reported in the Table. Some participants were unable to complete the entire imaging and functional testing protocol due to fatigue or time constraints. Only people with a viable macular scan and either the FDT or microperimetric test available were included. The Table also reports the number of participants for whom each variable/test was available. Eleven participants (10 healthy) had an intraocular lens implant. Three healthy participants and six diabetic patients had a PNS >1. The AL was measured for 495 participants and was derived through linear regression from the spherical equivalent for the remaining 10 participants (none of whom were pseudophakic). Despite a small difference in the average age composition, the two groups largely overlapped, and the main age clusters for diabetic patients were well represented in the healthy cohort. Microperimetry and FDT were available together for 412 participants (117 with DM). Thirteen participants were diagnosed with diabetes during the study because of their HbA1C value. Nineteen patients in this cohort had type I diabetes. Twenty-three participants had sings of early AMD, all in the no-DM cohort. The cp-RNFL scan was available for 386 people (118 with diabetes). All of these had performed the FDT test.

**Table.** Descriptive Statistics of the Analyzed Sample

Characteristic		No DM		DM		No. (No DM/DM)	
Age, y		61 (51, 66)		67 (58.25, 72)		371/134	
Sex, M:F, No.		208:163		88:46		371/134	
Duration of diabetes, y		—		0.2 (0.1, 0.43)		—/133	
HbA1C, %		5.57 (5.31, 5.89)		7.21 (6.52, 8.28)		271/118	
Spherical equivalent, diopters		0.38 (−0.75, 1.38)		0.38 (−0.62, 1.47)		369/134	
Axial length, mm		23.65 (22.96, 24.4)		23.49 (22.81, 24.15)		371/134	
BCVA (logMAR)		−0.08 (−0.14, 0)		−0.02 (−0.08, 0.04)		371/133	
PR-logCS (log)		1.65 (1.65, 1.65)		1.5 (1.5, 1.65)		370/133	
MAIA (MS, dB)		27.23 (26.09, 28.11)		26.56 (25.42, 27.88)		360/126	
FDT (global MD, dB)		−1.53 (−3.45, 0.17)		−2.32 (−4.43, −0.05)		306/125	
Age cluster, y	<30	30–39	40–49	50–59	60–69	70–79	>80
Healthy, No. (%)	36 (10)	23 (6)	26 (7)	84 (23)	155 (42)	45 (12)	2 (1)
Diabetes, No. (%)	3 (2)	8 (6)	3 (2)	23 (17)	47 (35)	45 (34)	5 (4)

Continuous variables are reported as median (interquartile range).

### Structural Metrics

**Figure 2.**
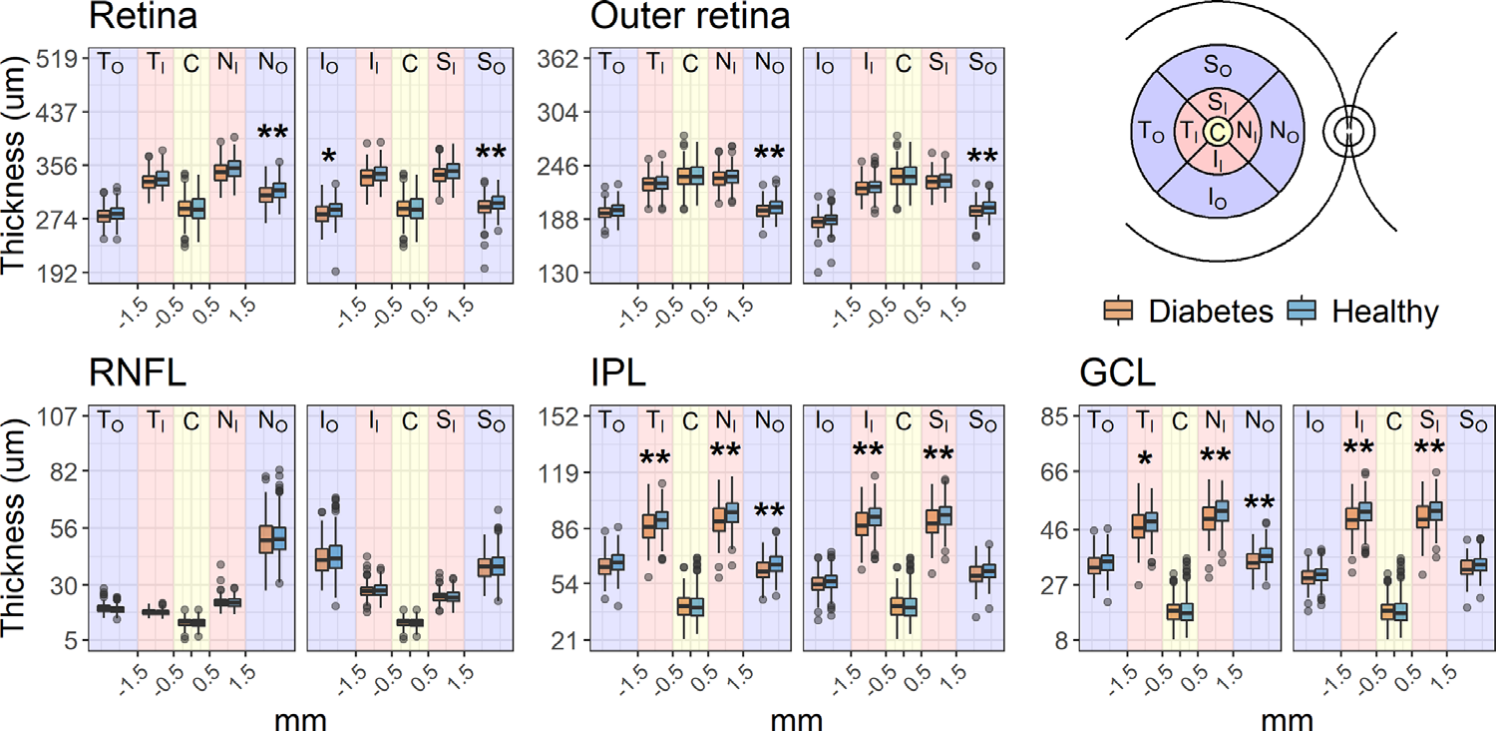
Boxplots of the average thickness values recorded for each ETDRS sector. The boxes enclose the interquartile range, and the whiskers extend to the 95% quantiles. *P* values were corrected for nine tests with the Bonferroni-Holm method for age-corrected comparisons. *P < 0.05. **P < 0.01.

Global average thickness across all sectors was significantly reduced in patients with DM for the whole retina (difference estimate [95% CIs]: –3.47 [–6.09 to –0.84] µm, *P* = 0.010), the GCL (–1.04 [–1.74 to –0.35] µm, *P* = 0.003), and the IPL (–1.89 [–3.09 to –0.69] µm, *P* = 0.002). No significant difference was found for the RNFL (0.11 [–0.52 to 0.74] µm, *P* = 0.730). The outer retina was generally thinner in the DM group, but this difference did not reach statistical significance (–1.65 [–3.37 to 0.07] µm, *P* = 0.061). The total macular log_10_(RGC count) was also significantly smaller in diabetic patients (0.011 [0.004 to 0.019] log_10_-unit reduction, *P* = 0.036). All comparisons were age-corrected by including age as a covariate, which was significantly negatively correlated with the thickness of all retinal layers (*P* < 0.001). Sector differences are reported in Figure 2. Significant differences (Bonferroni-Holm corrected *P* < 0.05) were found for the GCL and IPL in all 3-mm ring sectors and for the 6-mm nasal sector for all layers except the RNFL. In the DM cohort, the thickness of none of the layers was significantly correlated with either the HbA1C or the duration of diabetes. The average cp-RNFL, compensated for ocular magnification^45^ and corrected by age and axial length, was also significantly thinner in the DM cohort (–2.27 [–0.22 to –4.64] µm, *P* = 0.032).

### Functional Metrics

Diabetic people had a significantly lower PR-logCS (age-corrected estimated difference: –0.09 [–0.11 to –0.06], *P* < 0.001) and the BCVA (0.05 [0.03 to 0.07] logMAR, *P* < 0.001). There was no significant difference in microperimetric MS in the age-corrected comparison (–0.21 [–0.52 to 0.1] dB, *P* = 0.188). There was, however, a statistically significant difference in FDT cMD (–0.94 [–1.65 to –0.23] dB, *P* = 0.010) and FDT MD (–0.83 [–1.53 to –0.13], *P* = 0.021). A significant difference was also found for the age-corrected FDT cMS (–1.02 [–1.75 to –0.28], *P* = 0.007). In the DM cohort, none of the functional metrics were significantly correlated with either the HbA1C or the duration of diabetes.

#### Structure-Function Relationship

##### Global Structure-Function Relationship

**Figure 3.**
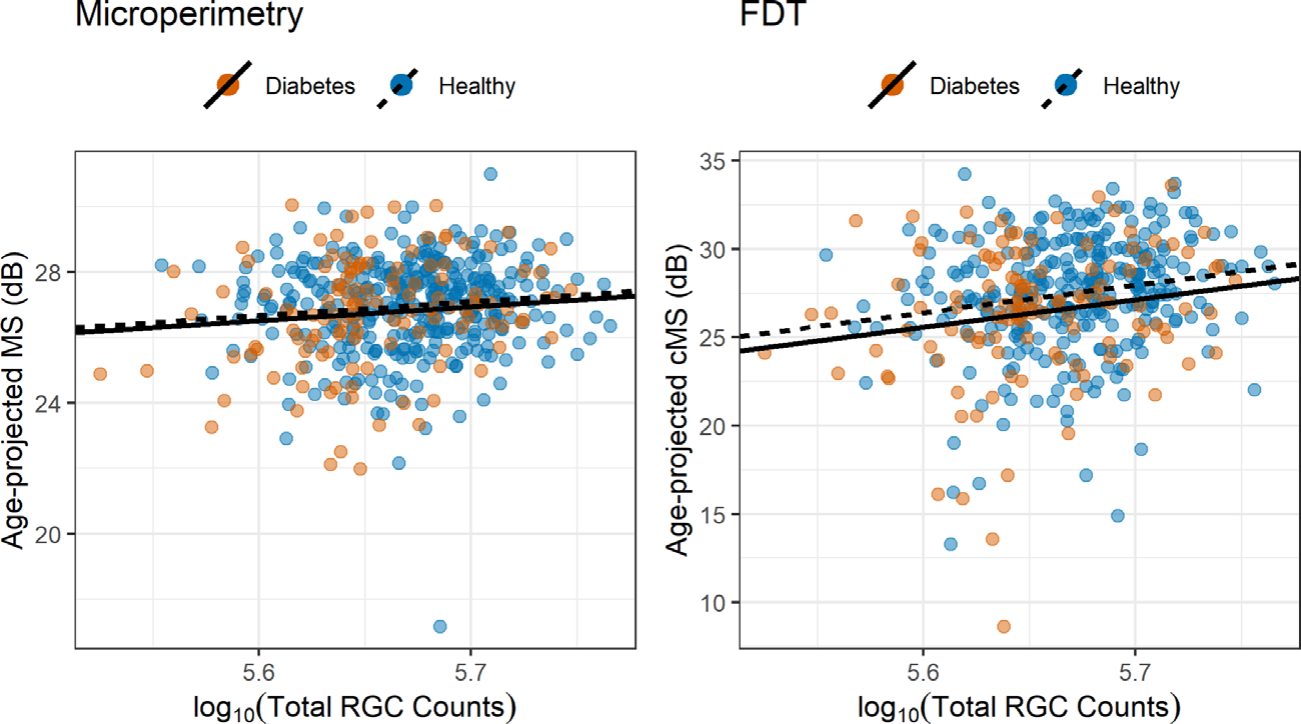
Scatterplot and regression lines for the global structure-function relationship. The regression lines have the same slope for both healthy and diabetic people. The total RGC count was calculated within the central 12 degrees from the fovea. The MS and cMS (12 central locations) are projected to the average age of the sample (58 years old).

A significant correlation with the total macular log_10_(RGC count) was found for the microperimetric MS (*P* = 0.0212) and for both central FDT metrics (cMD, *P* = 0.002; MS, *P* < 0.001). There was no significant difference in slopes between diabetic and healthy participants for either test (*P* = 0.055 for microperimetry; *P* = 0.885 for FDT cMD, *P* = 0.894 for FDT cMS). The slope was steeper for the FDT cMD (13.3 dB/log_10_(RGC count)) and FDT cMS (15.5 dB/log_10_(RGC count)) than microperimetry MS (4.3 dB/log_10_(RGC count)). However, this result needs to be interpreted in the context of the different definitions of the dB scale used by the two devices (see Discussion) and considering the small loss in RGC count effectively observed for diabetic people in this sample (see previous paragraph). A significant difference in intercepts was found for the FDT cMS (*P* = 0.030) but not for FDT cMD (*P* = 0.062) and microperimetry MS (*P* = 0.339). The relationships with age-corrected microperimetric MS and FDT cMS are shown in Figure 3. There was a significant correlation between the average cp-RNFL and the global FDT MS (0.04 dB/µm, *P* = 0.028). The relationship with the global MD, however, did not reach significance, despite being very similar in magnitude (0.03 dB/µm, *P* = 0.074). All comparisons with MS were corrected for age in the statistical model. The average cp-RNFL was also compensated for ocular magnification^45^ and corrected by AL.

##### Topographic Structure-Function Relationship

**Figure 4.**
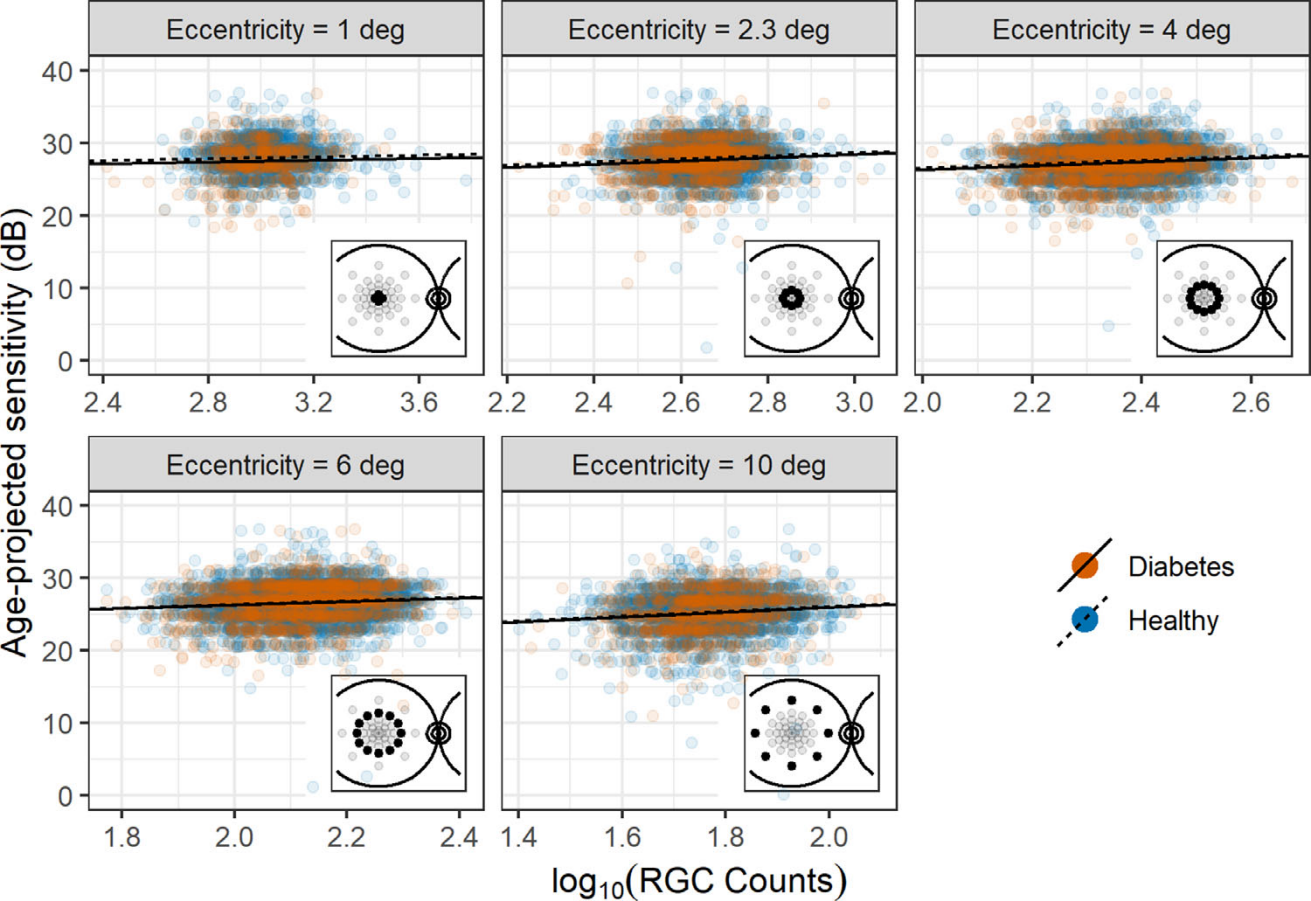
Scatterplot and regression lines for the topographic structure-function relationship for microperimetry. The regression lines have the same slope for both healthy and diabetic patients. Local counts account for RGC displacement. The microperimetric sensitivity is projected to the average age of the sample (58 years old).

The pointwise structure-function slopes for microperimetry were shallow (Fig. 4), as expected in the partial summation condition (see Appendix). The slopes were, however, all statistically significant (*P* < 0.05) except at 1 degree of eccentricity (*P* = 0.068). The only significant difference in intercepts between healthy and diabetic patients was found at 1 degree (*P* = 0.036). As expected, there was no significant difference in slopes between healthy and diabetic patients (*P* = 0.178).

**Figure 5.**
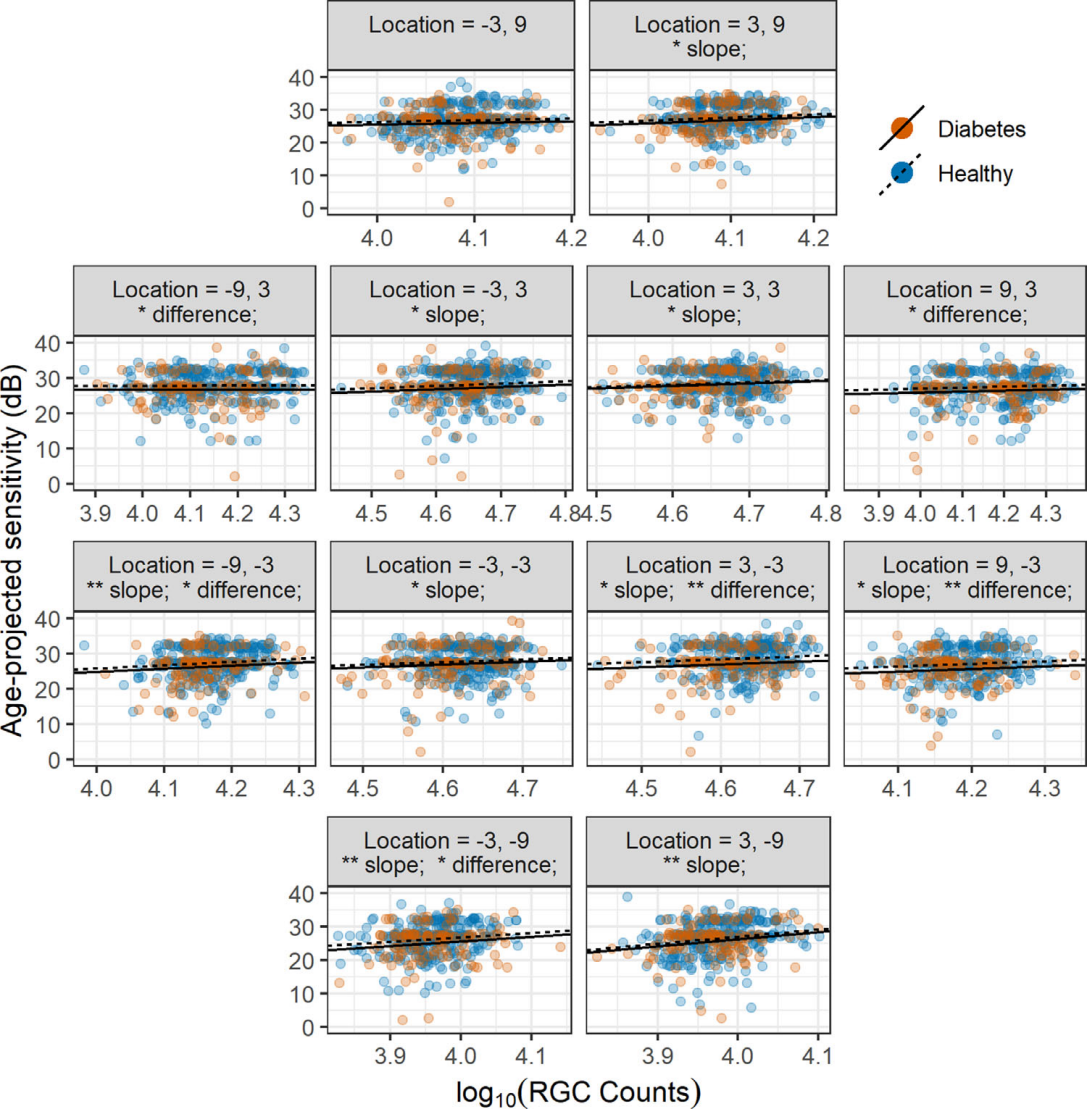
Scatterplot and regression lines for the topographic structure-function relationship for FDT. The regression lines have the same slope for both healthy and diabetic patients. Local counts account for RGC displacement. *P < 0.05. **P < 0.01. Difference = difference in intercepts between healthy and diabetic patients.

The pointwise structure-function slopes for FDT sensitivity values (Fig. 5) were also shallow. The significance for slopes and differences in intercepts between the two groups is reported in Figure 5 for each location. A significant difference in intercepts was found for five locations. A significant structure-function slope was found for nine locations. As expected, there was no significant difference in slopes between healthy and diabetic patients (*P* = 0.270).

## Discussion

We analyzed structural and functional data in a large number of patients with DM (*n* = 134) with no signs of DR and 371 healthy controls. Our results support the hypothesis of inner retinal loss prior to clinically evident vascular alterations in diabetes. Critically, we could test this hypothesis by excluding patients with DR, allowing us to isolate the effect of early neuronal loss. Another strength of our study is that the diagnosis of DM for many of the diabetic patients was fairly recent (Table). This constitutes an optimal condition to study neuronal loss in its earliest phase, suggesting that it might happen soon after or even before the clinical diagnosis of DM. Another novel important aspect of our analysis is that the results are framed in the context of an accepted neural model for perimetric stimuli, which allowed us to provide a mechanistic interpretation of the observed structure-function relationship rather than simply reporting statistical associations between structural and functional metrics. Such a model constitutes an accepted paradigm for glaucoma but has not been previously tested for early neuronal loss in diabetes.

### Structural Metrics

The largest significant reduction in retinal thickness was recorded for the GCL and IPL, with some mild, nonsignificant changes to the outer retina. This is in agreement with previous findings, showing thinning of the inner retina in patients with no or mild DR.^4^^,^^12^^–^^17^ Despite some local variations, the neural loss appeared mostly diffuse. However, most of the significant differences were found in sectors where the layer of interest is normally thicker, indicating a likely effect of a larger signal-to-noise ratio in areas where measurements are more robust and have more room for variation. Such a result is in agreement with Van Dijk et al.,^12^^,^^13^^,^^26^ who reported significant changes only in the perifoveal region. This can also explain the lack of observable differences in the RNFL, notoriously thin and difficult to measure in the macular region. Indeed, when the normally thicker cp-RNFL was analyzed, a significant, albeit small, loss was identified in patients with DM. It is important to note that retinal thinning is not the only structural change observed in patients with DM and minimal DR. A comprehensive analysis by Gerendas et al.^53^ showed GCL-IPL thickening and attributed it to initial diffuse swelling prior to the development of evident macular oedema. However, these findings pertained to patients with type 1 diabetes, including people with mild DR. Instead, in our study, we carefully focused our analysis on patients with no signs of DR to specifically examine evidence of neural degeneration. Our DM cohort only contained 19 patients with type 1 diabetes, too few to be analyzed separately. However, despite not being significant, this group showed an average thinning of the GCL compared to the healthy participants both in the raw (–0.47 µm) and age-corrected (–1.28 µm) estimates, in agreement with the general trend for the DM cohort. Nevertheless, this is an important aspect to consider when interpreting the structure-function relationship (discussed later).

### Functional Metrics

In agreement with previous results,^18^^–^^20^ PR-logCS was significantly reduced in diabetic patients (19% average age-corrected reduction in CS). A significant reduction was also observed in the BCVA, although the effect was smaller (12% average age-corrected increase in minimum angle of resolution). Such a small difference might explain why this parameter failed to show significant differences in previous reports.^18^^,^^20^

Importantly, we found a significant reduction in sensitivity with FDT perimetry in diabetic patients, confirming and expanding previous findings. Previous studies were mainly limited by the small sample size and the lack of a specific analysis of the macular region.^18^^,^^19^^,^^27^^–^^29^ In our analysis, we showed that the differences between healthy and diabetic patients are larger for the central locations (cMD) than the whole field (MD). This strengthens the evidence for neural damage, since tests of the central visual field are usually more reliable.^54^ We did not find any significant differences between healthy and diabetic patients in the age-corrected MS with microperimetry, despite a significant correlation with the RGC count. This can be explained by a more careful analysis of the structure-function relationship (see next paragraph). Of note, the MAIA, differently from the Matrix FDT perimeter, does not provide deviation values, hence the need for statistical correction for age. However, the same age-corrected model used for the central FDT MS still showed a strongly significant difference between the two groups, ruling out a lack of power in the statistical approach. Of course, one limitation of our data set is the lack of either the FDT or the microperimetry data for some of the participants. This could have been avoided by only analyzing complete data (76% of the overall sample, 88% of the diabetic cohort). However, we decided to include all participants who had performed at least one of the two perimetric tests to minimize the risk of bias and to maximize the power of our statistical analyses, conditioning our selection only on the presence of the macular OCT scan.

### Structure-Function Relationship

One core aspect of our analysis was the detailed study of the structure-function relationship, especially in the macular region. This is important in order to interpret our findings in the context of accepted neural models for perimetric responses. Unlike previous reports,^18^^,^^20^^,^^26^ we transformed the measured GCL thickness into an estimate of RGC counts, and this was a novel step. Such an approach allows a more direct interpretation of the functional findings in light of the observed structural changes. Both FDT and MAIA measurements showed a significant correlation with structural parameters in the global and topographical analyses. For the global parameters, the slopes were steeper (greater effect) for measurements from the FDT when compared to those from MAIA. However, it is important to keep in mind that the Matrix FDT equates one log_10_ step to 20 dB instead of 10 dB. To transform the FDT values in the same scale as microperimetry, it is sufficient to divide sensitivity and slopes by 2. This calculation brings the structure-function slope observed for the FDT cMS with the total central RGC count to 7.75 dB/log_10_(RGC count), much closer to the value observed for microperimetry (4.3 dB/log_10_(RGC count)), but still steeper.

Despite both tests showing a significant correlation with structural parameters, with global and local measurements, only the FDT was able to show a significant difference between diabetic and healthy participants. The lack of significant differences for microperimety can be explained by considering pointwise sensitivities and the effect of spatial summation on perimetric stimuli, and this is worthy of some discussion here and in the Appendix. Indeed, the relationship between the number of RGCs and perimetric sensitivity becomes very shallow if the number of stimulated RGCs is larger than a critical amount (conventionally >10^1.5^ for SAP stimuli^49^), reducing the ability of the test to discriminate early functional damage. This happens in the macular region for G-III stimuli (used in microperimetry) because of the high density of RGCs.^49^^,^^50^ Total summation conditions could be obtained for the macula by changing the size or the duration of the stimuli^49^^,^^55^ and would be particularly valuable for detecting the effect of early neural degeneration in diabetes. In fact, undersampling due to RGC loss is expected to have a greater effect on sensitivity for small test targets compared to large test targets (see Appendix). However, FDT was able to discriminate between the two groups regardless of this limitation. Although such simple reasoning is more difficult to apply to FDT stimuli, the even larger stimulus size is likely to produce partial summation (see Appendix). One explanation for this difference is that FDT might be able to detect early cell dysfunction occurring in diabetic patients, in addition to the changes explained by pure structural loss. This is concordant with the finding that a significant difference in the intercepts was detected in the structure-function relationship for FDT metrics (with no significant differences in slope), effectively highlighting a residual functional defect in diabetic patients unexplained by structural changes. This residual defect could be the consequence of concomitant changes in the functionality of the outer retina. However, given the lack of significant thinning of the outer layers, this seems unlikely for our data set. Of course, such a difference in intercepts could also be explained by the limitations of the structural OCT measurements. One key assumption in our structure-function analyses is that changes in the measured thickness values accurately represent the loss of neural tissue. This is known not to be the case and is one of the reasons for the floor effect in structural measurements, especially with more advanced damage.^47^^,^^56^ For example, our quantification of RGCs assumes that cellular density within a given volume of tissue remains constant and the change in RGCs is accurately reflected by the change in volume. Moreover, as previously mentioned, inner retinal tissue thickening has also been described^53^ in diabetic patients, likely due to subtle swelling of the neural tissue. This would make our assumption of constant density unreliable. However, it is unlikely for these factors to have played a major role in our analyses, given the absence of eyes with DR and the relatively early loss of inner retinal tissue, far from the floor effect. Indeed, such inaccuracies should have caused a significant difference in intercepts between the two groups also for microperimetry, which was not seen. This opens up potential applications of complex stimuli to more accurately investigate inner retinal damage in diabetes. However, in other reports, traditional SAP was also shown to be effective in detecting retinal dysfunction in diabetes^18^^,^^28^^–^^31^ and performed similarly to FDT when compared directly.^18^ The recent introduction of wide-field photopic white-on-white perimeters equipped with fundus tracking technology^57^ might combine the accuracy of microperimetry with the benefit of traditional SAP. The obvious advantage of circular stimuli is that, not having to accommodate for patterns, they can be designed to be arbitrarily localized (small), potentially increasing spatial precision. However, as mentioned earlier, the characteristics of the stimulus (duration/size) should ideally be optimized to detect fine changes in the macular region (this point is further expanded in the Appendix).

### Effect of Disease Duration and HbA1C

We could not find any significant correlations of the structural or functional parameters with either the duration of the disease or the percentage HbA1C in diabetic patients. The measured impact of these factors on neuronal damage has been variable across different reports.^13^^,^^21^^,^^53^^,^^58^ In our study population, the average duration of the disease was short. This was expected from our selection criteria, since patients with type 2 diabetes and no DR are likely to have only been recently diagnosed. This also means that the recorded duration is unlikely to accurately reflect the actual time course of the disease. Longer durations were recorded for patients with type 1 diabetes, a small fraction of our sample.

A similar consideration can be made for the HbA1C, since the value measured in our cohort is representative of the metabolic control under treatment, with little connection to the metabolic imbalance that would have determined the initial neural damage. One limitation of this analysis was the fact that the HbA1C was not available for all participants (88% of diabetic patients and 73% of healthy participants). This constitutes a limitation also for the exclusion of type 2 DM in the healthy cohort. However, only 37 of the healthy participants (10% of the overall healthy cohort) for whom HbA1C was not measured were older than 40 years of age and therefore at reasonable risk of having undetected type 2 DM. Thus, such a misclassification might have reduced the observed differences between the two cohorts in our data set but is unlikely to have produced a large effect.

## Conclusions

Our data provide structural and functional evidence to support the hypothesis of neuronal damage in DM, prior to clinically evident vascular changes, in a large cohort of diabetic patients and healthy controls. However, most of these modifications are subtle and difficult to detect. The macular region has the potential to be the optimal “ground” to integrate structural and functional information for early detection of neural degeneration. Although these changes are too small to directly impact on patients’ vision, their detection is clinically meaningful, as it could help predict the insurgence of clinically evident vascular alterations.^5^ However, functional tests should be optimized to better probe the central visual field, taking the effect of neural summation into account, for example. Future investigations with better designed functional tests are needed to assess the clinical effectiveness of structure-function integration to detect early neural damage in diabetes. Our data also confirm that a simple measurement of BCVA might be insufficient to fully characterize the changes in visual function observed in diabetes and that perimetric tests should be considered by researchers investigating diabetic neuronal damage. It is also important to highlight that clinical studies such as this cannot entirely rule out the presence of preexisting microalterations of the retinal vasculature as a primary source of neuronal damage, since only clinically evident vascular alterations can be excluded in patients. Further structure-function analyses including parameters from OCT-angiography scans might help shed light into this aspect and will be the subject of future work.

## Figures and Tables

**Figure A1.**
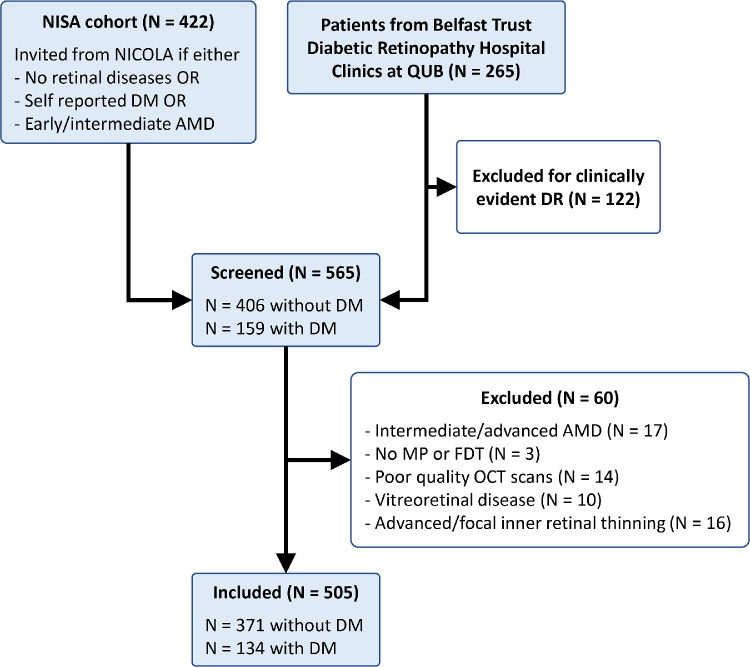
Flowchart of the selection steps detailed in the methods.

**Figure A2.**
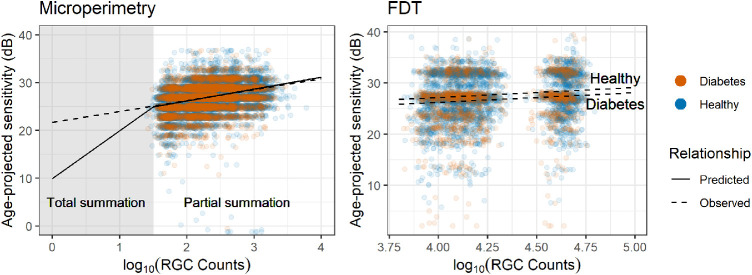
Observed structure-function relationship compared to the theoretical framework of spatial summation. The observed relationship for the FDT is plotted as two separate lines to reflect the significant difference in intercepts between the diabetic and healthy participants. Sensitivity is projected to the average age of the sample (58 years old).

**Figure A3.**
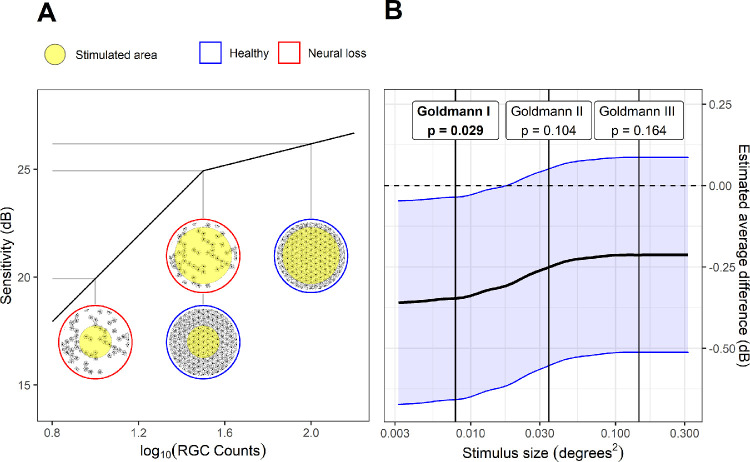
Example of how the microperimetric test could be modified to improve detection of neural damage. (**A**) Differences between perimetric responses from a healthy and degraded RGC mosaic are amplified by smaller stimuli that operate in total summation conditions. The *gray lines* connect the number of RGCs stimulated in each condition with the predicted sensitivity. Their difference can be read on the vertical axis. (**B**) This principle might have provided different results for microperimetry in this study; the *black line* represents the estimated average difference in sensitivity with different stimulus sizes, according to the model; the *blue shaded area* encloses the 95% confidence intervals for the difference, estimated from a linear mixed model. The vertical lines indicate the size of typical Goldmann stimuli. The estimated difference was significant with a Goldmann I stimulus (*P* values reported at the top).

## Appendix

### Flowchart of the Selection Steps

The flowchart in Figure A1 reports the selection steps applied to the initial samples. The criteria for patients to be invited for the NISA study were based on the information collected at the time of the NICOLA study. For example, people with early AMD during the NICOLA study might have converted to intermediate/advanced AMD and therefore later excluded from this analysis. No such prior information was available for the diabetic cohort from the Belfast Trust Diabetic Retinopathy Hospital Clinics at QUB.

### Spatial Summation of Perimetric Stimuli

Spatial summation describes how sensory systems combine input from multiple channels to produce the final psychophysical sensation (in this case, perimetric sensitivity). For simple circular perimetric stimuli, such as in the case of the MAIA, the number of channels is often equated to the number of RGCs being stimulated.^49^^,^^59^ This number can change because of the size of the stimulus or the local density of RGCs.^49^^,^^55^^,^^59^ In SAP, the relationship between the log_10_(RGC count) and sensitivity in dB has a slope of 10 up to a certain RGC count (total or complete summation), after which the slope becomes much shallower, usually 2.5 (partial summation). In traditional SAP with photopic background illumination (10 cd/m^2^) and 200-ms round stimuli, the break point is conventionally located at 10^1.5^ RGCs.^49^ Although using a mesopic background (1.27 cd/m^2^), such as in the MAIA, could change the location of the breakpoint,^60^^,^^61^ large differences are not expected for changes of less than 1 log_10_ unit in background illumination, as in this case. In fact, the theoretical framework is confirmed by our experimental data. Figure A2, left panel, shows the relationship between the log_10_(RGC count) and microperimetric sensitivity. The empirical slope fitted using the overall data set yielded a value of 2.29 (95% CI, 2.22–2.37) dB/log_10_(RGC count), remarkably close to the theoretical prediction. Indeed, even for the most peripheral locations, the number of stimulated ganglion cells was mostly beyond the critical 10^1.5^. Of note, the empirical slope was fitted using a linear mixed model with random intercepts to account for correlated measures from the same eye and with age as a covariate to account for the aging effect beyond the normal loss of RGCs. In this framework, significant differences in intercepts between the diabetic patients and healthy controls would indicate a sensitivity loss unexplained by the structural loss, for example, in the case of dysfunction. This was not observed for microperimetric stimuli.

Such a theoretical framework can be applied for FDT stimuli, although it is harder to interpret due to their complex features.^41^ Nevertheless, the much larger stimulus size is likely to produce partial summation conditions. Indeed, the observed slope was 2.29 (95% CI, 1.97–2.62) dB/log_10_(RGC count). This is, however, much shallower than microperimetry, considering that for the Matrix FDT, one log_10_ step corresponds to 20 dB instead of 10 dB, leading to slope estimates that are doubled in value. In this case, as for the main analysis, there was a significant difference in the intercept between the two groups (*P* = 0.0105), and this is represented in Figure A2, right panel.

Figure A3 shows how different results could be obtained by performing the microperimetric test in total summation conditions, for example, by reducing the stimulus size. For this calculation, we scaled the log_10_(RGC count) estimated for Goldmann III stimuli to other stimulus sizes. We then calculated the expected sensitivity for the corresponding number of stimulated RGCs according to the model and added the residuals calculated from the real data. The estimated average difference between the two groups (and its standard error) was then calculated by combining the values at different locations using a linear mixed model. As expected, the difference became larger for smaller stimulus sizes (Fig. A3B) and reached significance with a Goldmann I. This calculation does not account for possible changes in the variability of the perimetric response that could be introduced when testing with smaller stimulus sizes. However, this is not an intrinsic limitation of the test, since strategies can be devised to maintain constant variability at different sensitivities.^62^ Moreover, the breakpoint location might change based on adaptation conditions and background luminance,^60^^,^^61^ but a Goldmann I stimulus is expected to be within the range of total summation in the macula even for mesopic testing conditions.^63^
